# Repositioning Canagliflozin for Mitigation of Aluminium Chloride-Induced Alzheimer’s Disease: Involvement of TXNIP/NLRP3 Inflammasome Axis, Mitochondrial Dysfunction, and SIRT1/HMGB1 Signalling

**DOI:** 10.3390/medicina60111805

**Published:** 2024-11-03

**Authors:** Hemat A. Elariny, Ahmed M. Kabel, Heba Mohammed Refat M. Selim, Azza I. Helal, Doaa Abdelrahman, Hany M. Borg, Mennatallah A. Elkady, Lamees M. Dawood, Mohamed F. El-Badawy, Haifa Faisal A. Almalawi, El-Shaimaa A. Arafa, Shuruq E. Alsufyani, Hany H. Arab

**Affiliations:** 1 Department of Pharmacology and Toxicology, College of Pharmacy, University of Hail, Hail 55476, Saudi Arabia; hem.mohammed@uoh.edu.sa; 2 Department of Pharmacology and Toxicology, Faculty of Pharmacy, Al-Azhar University, Cairo 35527, Egypt; 3 Pharmacology Department, Faculty of Medicine, Tanta University, Tanta 31527, Egypt; ahmed.kabal@med.tanta.edu.eg (A.M.K.); mennah.elkady@med.tanta.edu.eg (M.A.E.); 4 Department of Pharmaceutical Sciences, College of Pharmacy, AlMaarefa University, P.O. Box 71666, Riyadh 11597, Saudi Arabia; hmustafa@um.edu.sa; 5 Department of Histology and Cell Biology, Faculty of Medicine, Kafrelsheikh University, Kafr El-Shaikh 33516, Egypt; aza_hlal2014@med.kfs.edu.eg; 6 Internal Medicine Department, College of Medicine, Princess Nourah Bint Abdulrahman University, P.O. Box 84428, Riyadh 11671, Saudi Arabia; 7 Physiology Department, Faculty of Medicine, Kafrelsheikh University, Kafr El-Shaikh 33516, Egypt; hany_borg@med.kfs.edu.eg; 8 Medical Biochemistry Department, Faculty of Medicine, Tanta University, Tanta 31511, Egypt; lamees.dawood@med.tanta.edu.eg; 9 Microbiology and Immunology Department, Faculty of Pharmacy, University of Sadat City, Sadat City 32897, Egypt; mohamed.elbadawy@fop.usc.edu.eg; 10 King Faisal Medical Complex, Taif Health Cluster, Taif 26513, Saudi Arabia; haifaisal9080@gmail.com; 11 College of Pharmacy and Health Sciences, Ajman University, Ajman 346, United Arab Emirates; e.arafa@ajman.ac.ae; 12 Center of Medical and Bio-Allied Health Sciences Research, Ajman University, Ajman 346, United Arab Emirates; 13 Department of Pharmacology and Toxicology, College of Pharmacy, Taif University, P.O. Box 11099, Taif 21944, Saudi Arabia; s.alsofyani@tu.edu.sa (S.E.A.); h.arab@tu.edu.sa (H.H.A.)

**Keywords:** canagliflozin, aluminium chloride, Alzheimer’s disease, sirtuin-1, behavioural changes, rats

## Abstract

*Background and Objectives:* Alzheimer’s disease (AD) is the most common neurodegenerative disorder in the world. Due to failure of the traditional drugs to produce a complete cure for AD, the search for new safe and effective lines of therapy has attracted the attention of ongoing research. Canagliflozin is an anti-diabetic agent with proven efficacy in the treatment of neurological disorders in which mitochondrial dysfunction, oxidative stress, apoptosis, and autophagy play a pathophysiological role. Elucidation of the potential effects of different doses of canagliflozin on AD induced by aluminium chloride in rats and exploration of the molecular mechanisms that may contribute to these effects were the primary objectives of the current study. *Materials and Methods:* In a rat model of AD, the effect of three different doses of canagliflozin on the behavioural, biochemical, and histopathological alterations induced by aluminium chloride was assessed. *Results:* Canagliflozin administered to aluminium chloride-treated animals induced dose-dependent normalisation in the behavioural tests, augmentation of the antioxidant defence mechanisms, inhibition of TXNIP/NLRP3 inflammasome signalling, modulation of the SIRT1/HMGB1 axis, interference with the pro-inflammatory and the pro-apoptotic mechanisms, and restoration of the mitochondrial functions and autophagy in the hippocampal tissues to approximately baseline values. In addition, canagliflozin exhibited an interesting dose-dependent ability to repress aluminium chloride-induced histopathological changes in the brain. *Conclusions:* The effects of canagliflozin on oxidative stress, mitochondrial functions, inflammatory pathways, and autophagy signals may open new gates towards the mitigation of the pathologic features of AD.

## 1. Introduction

Neurodegenerative diseases are pathological disorders characterised by profound neuroinflammation, synaptic dysfunction, and profound effects on the functions of the nervous system [1]. Of these, Alzheimer’s disease (AD) is considered the most prevalent form around the world [2]. The hallmark of AD includes excessive β-amyloid peptide deposition together with appearance of multiple senile plaques in the cerebral tissues [3]. These changes are usually accompanied with degenerative changes in the brain neurons together with massive synaptic loss [4]. These pathologic events clinically manifest as memory defects, cognitive dysfunction, behavioural changes, and motor incoordination [5].

As of today, no single etiological factor can be incriminated to be responsible for the development of AD [6]. However, massive neuroinflammation with subsequent effects on neuronal functions was considered as the main precipitating factor of the functional disabilities encountered in AD [7]. In addition, perturbation of the redox state with significant increases in reactive oxygen species (ROS) production, effects on the mediators of autophagy, and augmentation of the apoptotic signalling pathways were suggested to play a crucial role in the pathognomonic events of AD [8].

Over the years, several animal models have been utilised for exploration of the molecular mechanisms that might underlie AD [9]. These include chemically induced, genetically manipulated, high-fat-diet-induced, fructose-induced, lesion-induced, and in vitro-induced models [10]. Chemically induced AD can be elicited in rodents by administration of streptozotocin, amyloid β42, colchicine, scopolamine, atropine, and aluminium chloride [11]. Of these, intraperitoneal injection of aluminium chloride is a commonly used model that represents most of the pathognomonic features of AD [12]. This might be attributed to the ability of aluminium chloride to easily pass the blood–brain barrier through its high affinity for the transferrin receptors in the brain, mostly in the hippocampus and the frontal cortex [13]. Once accumulated, aluminium chloride was reported to have an outstanding ability to enhance ROS production and to induce a state of profound neuroinflammation with significant deposition of amyloid β in the cerebral tissues and enhancement of tau phosphorylation [14]. Interestingly, administration of aluminium chloride by the intraperitoneal route is easy and avoids the invasive intracerebroventricular administration of other chemicals such as streptozotocin and amyloid β [11].

Canagliflozin is an agent that inhibits sodium–glucose cotransporter 2 (SGLT2), thus contributing to the amelioration of hyperglycaemia in type 2 diabetes mellitus [15]. Ongoing research has proven the potent neuroprotective effects of canagliflozin against the vast majority of neurological illnesses in different animal models [16]. These effects may originate from the mitigating effect exerted by canagliflozin on ROS production as a consequence of neurotoxic stimuli and its inhibitory effects on the expression of the mediators involved in the inflammatory cascade [17]. This in addition to the effects of canagliflozin on autophagy/apoptosis balance might raise the need for exploration of the potential effects of canagliflozin on AD [18]. This work was a trial to explore the potential effects of administration of different doses of canagliflozin on a rat model of AD induced by aluminium chloride injection and to uncover the possible molecular mechanisms that may contribute to these effects.

## 2. Materials and Methods

### 2.1. Ethical Statements

The design of the experiments and the methods of handling the animals in the current work followed the ARRIVE guidelines. The present study attained ethical approval from the Research Ethics Committee of Faculty of Medicine, Tanta University, Egypt (Code 36264PR287/8/23).

### 2.2. Drugs and Chemicals

Aluminium chloride was purchased from Tokyo Chemical Industry (Tamil Nadu, India) Pvt. Ltd. (CAS number 7446-70-0, Product Number: A1831). Canagliflozin was provided by Santa Cruz Biotechnology, Inc., Dallas, TX, USA (CAS number 842133-18-0). Carboxymethyl cellulose (CMC) was supplied by Sigma-Aldrich Chemical Co., St. Louis, MO, USA (CAS number 9004-32-4). Normal saline (0.9% sodium chloride solution) was used for dissolving aluminium chloride. Canagliflozin was suspended in 0.5% CMC solution. Other chemical materials used in the current work were of analytical grade and were provided by Shanghai Korain Biotech Co., Shanghai, China.

### 2.3. The Experimental Design

**Figure 1:**
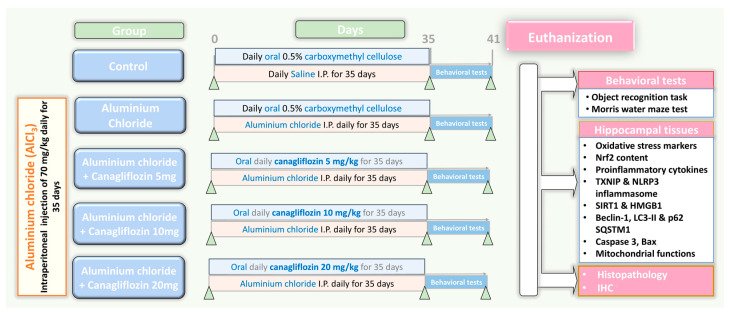
A schematic representation of the experimental design of the study.

Fifty sexually mature male Sprague Dawley rats weighing about 200–250 g were kept in special metabolic cages (temperature 23 ± 3 °C, relative humidity 55 ± 10%, 12 h light/dark cycles) with free access to food and water ad libitum for two weeks before being subjected to any experiments to make sure that they were properly acclimatised. Thereafter, a research assistant who was unaware of the protocol of the study randomly categorised rats into five equal groups (10 animals/group) as demonstrated in Figure 1 as follows: control animals received daily intraperitoneal injection of 0.5 mL of normal saline solution concomitantly with 1 mL of 0.5% CMC solution daily by oral gavage for 35 days; the aluminium chloride group received daily I.P. injection of aluminium chloride (70 mg/kg) [19] concomitantly with 1 mL of 0.5% CMC solution daily by oral gavage for 35 days; canagliflozin (5 mg/kg/day) was administered orally to the aluminium chloride + canagliflozin 5 mg group [17]; 10 mg/kg/day canagliflozin was given orally to the aluminium chloride + canagliflozin 10 mg group [17]; and in the aluminium chloride + canagliflozin 20 mg group, aluminium chloride-injected animals received canagliflozin in a dose of 20 mg/kg daily orally [20]. Canagliflozin was administered daily by oral gavage concomitantly with aluminium chloride injection for 35 days.

### 2.4. Determination of the Behavioural Changes in the Studied Animals

Starting from the 36th day of the experiment, behavioural tests were performed to assess the memory changes and the cognitive functions of rats from the different groups. These included the Morris Water Maze test and object recognition test.

#### 2.4.1. Morris Water Maze Test

A circular water-filled container of 50 cm depth with an escape platform which was put 1 cm below the water surface was used for evaluation of memory and cognition of the tested animals. This test had two stages; the training and the testing stages. For training, every rat had three trials to find the platform per day for 5 consecutive days. The testing stage was carried out on the 6^th^ day where the platform was removed and the animal was allowed to swim in the container. A video tracking system was utilised to record the swimming speed, the time spent in the target quadrant, and the path length for each animal [21].

#### 2.4.2. Object Recognition Test

This test utilised a square black-painted wooden box which has constant illumination. In this box, 2 different wooden block toys were put and represented familiar objects to the animals. This test was executed via 3 different stages, namely habituation, training, and testing. For habituation, rats were put in the wooden box for 10 min to be adapted. After 24 h, two familiar objects were put in the box and rats were allowed to explore them for 8 min. In the testing stage, two objects—one novel and one familiar—were put in the box and the animals were given a chance to explore these objects for 8 min. The sniffing time taken by each animal for each object was recorded via a video tracking system. The discrimination index was determined by subtracting the time spent by the animal on the familiar object from the time spent on the novel object. The result was divided by the sum of time spent by the animal on the novel object and the time spent on the familiar one [22].

After assessment of the behavioural changes, rats were euthanized under isoflurane anaesthesia and the hippocampus was excised. Specimens of the hippocampal tissues were homogenised, the homogenate was centrifuged at 3000 rpm for 15 min, and the resulting supernatant was the working material in which the biochemical parameters were assessed. In addition, other parts of the hippocampus were processed for further histopathological examination.

### 2.5. Assessment of the Impact of Different Doses of Canagliflozin on Aluminium Chloride-Induced Changes in Nuclear Factor Erythroid 2-Related Factor 2 (Nrf2) Content and the Redox State of the Hippocampal Tissues

Tissue Nrf2 content was assessed using kits purchased from Shanghai Korain Biotech Co., Ltd., Shanghai, China (Code number E1083Ra). Tissue malondialdehyde (MDA) content and the total antioxidant capacity (TAC) were quantified using kits obtained from Wuhan Fine Biotech Co., Wuhan, Hubei, China (Catalogue numbers ER1878 and K025, respectively). Kits supplied by Elabscience, Houston, TX, USA, were utilised to assay glutathione reductase (GR) levels in the hippocampal tissues (Catalogue number E-BC-K099-S). Additionally, the levels of glutathione peroxidase (GPx) in the hippocampal tissues were quantified using kits purchased from CUSABIO, Houston, TX, USA (Catalogue number CSB-E12146r).

### 2.6. Determination of the Hippocampal Tissue Tumour Necrosis Factor Alpha (TNF-α), Interleukin 1 Beta (IL-1β), and IL-6

Boster Biological Technology, Pleasanton, CA, USA, was the provider of ELISA kits used for quantification of TNF-α levels in the hippocampal tissues (Catalogue number EK0526). The hippocampal tissue content of IL-1β and IL-6 was quantified using kits supplied by Abcam, Waltham, MA, USA (Catalogue numbers ab100768 and ab234570, respectively). The instructions included in the datasheet were used for proper determination of these parameters.

### 2.7. Assessment of the Hippocampal Tissue Levels of Thioredoxin-Interacting Protein (TXNIP), Nuclear Factor Kappa B (NF-κB) p65, and Nucleotide-Binding Domain-like Receptor Family, Pyrin Domain-Containing 3 (NLRP3) Inflammasome

The hippocampal tissue levels of TXNIP and NF-κB p65 were determined using ELISA kits supplied by Biorbyt Ltd., Cambridge, UK (Catalogue numbers orb1210541 and orb410907, respectively). Kits purchased from Abcam, Waltham, MA, USA, represented the tools that were utilised for quantification of tissue NLRP3 inflammasome levels (Catalogue number ab277086).

### 2.8. Quantification of the Hippocampal Tissue Levels of High-Mobility Group Box 1 (HMGB1) and Sirtuin-1 (SIRT1)

Novus Biologicals, LLC, Centennial, CO, USA, was the provider of the kits that were utilised for assessment of the hippocampal tissue content of HMGB1 (Catalogue number NBP3-06661). CliniSciences, Nanterre, France, was the source of ELSA kits used for quantification of tissue SIRT1 levels (Catalogue number orb780035-48).

### 2.9. Determination of the Hippocampal Tissue Levels of the Autophagy Markers

Tissue beclin-1 was assayed using kits supplied by Biorbyt Ltd., Cambridge, CB4 0WY, UK (Catalogue number orb1211726). Tissue p62 SQSTM1 levels were quantified using sandwich ELISA kits purchased from LSBio, Shirley, MA, USA (Catalogue number LS-F74536). Cell Biolabs, Inc., San Diego, CA, USA, was the provider of the kits used for the determination of tissue LC3-II levels (Catalogue number CBA-5116).

### 2.10. Assessment of the Hippocampal Tissue Levels of Caspase 3 and Bax

CliniSciences, Nanterre, France, was the source of ELISA kits used for the determination of tissue caspase 3 (Catalogue number orb410890). The tissue levels of Bax were quantified using sandwich ELISA kits purchased from Wuhan Fine Biotech Co., Wuhan, Hubei, China (Catalogue number ER0512).

### 2.11. Determination of the Mitochondrial Functions in the Hippocampal Tissues

Differential centrifugation was selected as the suitable method for isolation of the mitochondria according to Olivera and Meigs [23]. The tissue levels of mitochondrial ATP and the activity of the mitochondrial complex I (NADH-Ubiquinone oxidoreductase enzyme) were determined using kits provided by Abcam, Waltham, MA, USA (Catalogue numbers ab83355 and ab109721, respectively). The mitochondrial transmembrane potential was quantified according to Maity et al. [24].

### 2.12. Microscopic Evaluation of the Pathological Changes in the Hippocampal Tissues

Specimens of the extracted hippocampal tissues were immediately fixed in neutral buffered formalin (10%) and then immersed in paraffin to form paraffin blocks which were cut with a microtome into slices of 5 µm thickness. After that, these slices were stained with hematoxylin and eosin (H&E) and examined using light microscopy (Olympus, Tokyo, Japan).

### 2.13. Evaluation of the Extent of the Immunohistochemical Positive Expression of B-Cell Lymphoma-2 (BCL-2) Protein in the Hippocampal Tissues

The hippocampal tissue specimens were immunostained using an anti-BCL-2 monoclonal antibody (Boster Biological Technology, Pleasanton, CA, USA, catalogue number M00040-3). The tissue specimens were incubated at 4 °C overnight (1:20 dilution). Later on, they were rinsed in phosphate-buffered saline (PBS) for 15 min and the secondary antibodies which were diluted in phosphate-buffered saline (1:200 dilution) were applied. Then, the tissue sections were incubated for 45 min at 25 °C after which counterstaining with hematoxylin was performed followed by dehydration and examination under a light microscope (Olympus, Tokyo, Japan). The intensity of the brown staining was taken as an indicator of BCL-2 immunoreactivity, which was quantified using ImageJ software (ImageJ version 1.53a, NIH, Bethesda, MD, USA).

### 2.14. Data Evaluation

The results obtained from the present study were reported as means and standard deviations (SDs). Analysis of data was performed using the statistical tools of GraphPad Prism, version 7 (GraphPad Software, LLC, San Diego, CA, USA). For assessment of the normal distribution of data obtained, the Shapiro–Wilk normality test was utilised. Levene’s test was used to test the homogeneity of variances. Comparison of the different groups to each other was carried out with the means of a one-way analysis of variance (One-way ANOVA) followed by a post hoc Tukey test for groups with equal variances, and Welch ANOVA followed by a Games Howell test for groups with unequal variances when assessed by a Bartlett test. The level of statistical significance was indicated by a *p*-value less than 0.05.

## 3. Results

### 3.1. Canagliflozin Dose-Dependently Mitigated the Behavioural Changes Induced by Aluminium Chloride Injection

**Figure 2:**
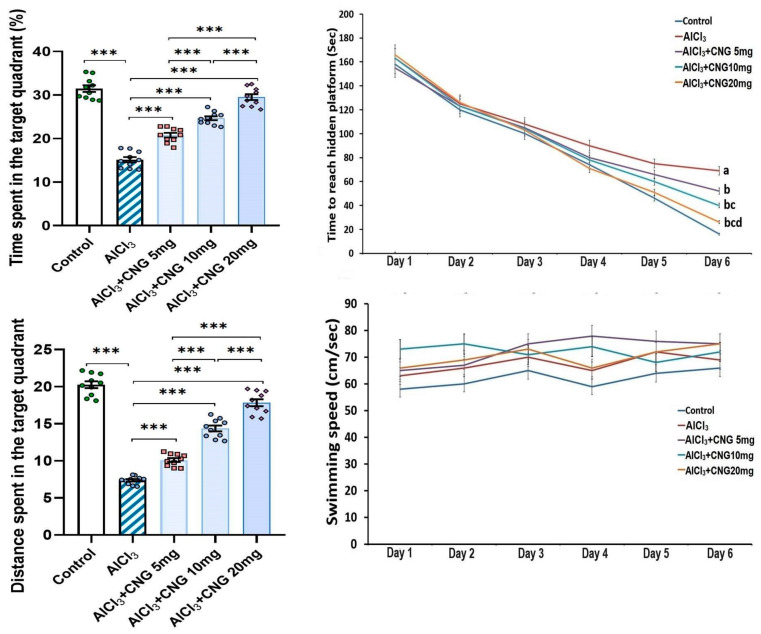
Effect of the different doses of canagliflozin (CNG) on Morris Water Maze test in rats injected with aluminium chloride (mean ± SD). ^a^ Significant versus the control group; ^b^ significant versus aluminium chloride-injected group; ^c^ significant versus aluminium chloride-injected rats treated with canagliflozin 5 mg/kg; ^d^ significant versus aluminium chloride-injected rats treated with canagliflozin 10 mg/kg; *** = *p* < 0.001. AlCl_3_, aluminium chloride; CNG, canagliflozin.

As depicted in Figure 2, the mean swimming speed in the Morris Water Maze test did not show any statistical difference between any of the studied groups. However, a significant prolongation of the time taken to reach the hidden platform, associated with a significant decline in the time and the distance spent in the target quadrant, was detected in the aluminium chloride-treated group when compared to the control group. Amazingly, treatment with canagliflozin elicited a dose-dependent decrement in the time taken to reach the hidden platform together with a significant dose-dependent lengthening of the time and the distance spent in the target quadrant relative to rats treated with aluminium chloride alone.

**Figure 3:**
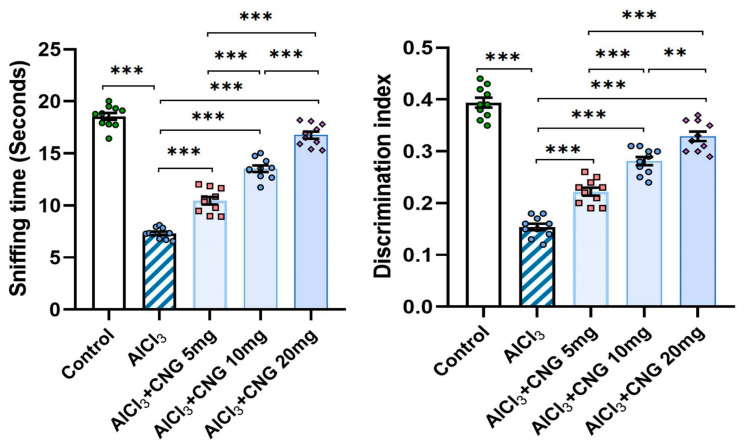
Effect of the different doses of canagliflozin (CNG) on the sniffing time and the discrimination index in rats injected with aluminium chloride (mean ± SD); ** = *p* < 0.01, *** = *p* < 0.001. AlCl_3_, aluminium chloride; CNG, canagliflozin.

Figure 3 shows that animals injected with aluminium chloride alone showed a significant diminution of the sniffing time and the discrimination index relative to the control animals. Interestingly, animals treated with canagliflozin exhibited a dose-dependent increase in the sniffing time and the discrimination index in comparison to the animals treated with aluminium chloride alone.

### 3.2. Canagliflozin Dose-Dependently Abrogated the Effect of Aluminium Chloride Injection on the Redox State and Nrf2 Content of the Hippocampal Tissue Specimens

**Figure 4:**
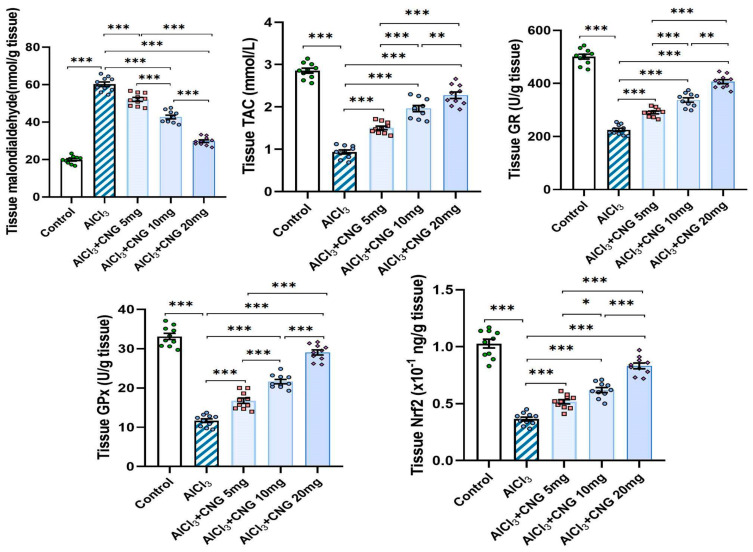
Effect of the different doses of canagliflozin on the redox status and Nrf2 content of the hippocampal tissues (mean ± SD); * = *p* < 0.05, ** = *p* < 0.01, *** = *p* < 0.001. AlCl_3_, aluminium chloride; CNG, canagliflozin.

As illustrated in Figure 4, administration of aluminium chloride induced a significant perturbation of the redox state of the hippocampal tissues. This perturbation was evidenced by a significant elevation of tissue MDA associated with a significant decrement in tissue TAC, GR, GPx, and Nrf2 relative to the control rats. Treatment with canagliflozin showed an outstanding dose-dependent capacity to decrease tissue MDA levels and restore Nrf2 content and the antioxidant defence mechanisms of the hippocampal tissues to approximate the control values.

### 3.3. Canagliflozin Dose-Dependently Counteracted the Effect of Aluminium Chloride Administration on TXNIP/NF-κB/NLRP3 Inflammasome Signalling and the Inflammatory Cascade in the Hippocampal Tissue Specimens

**Figure 5:**
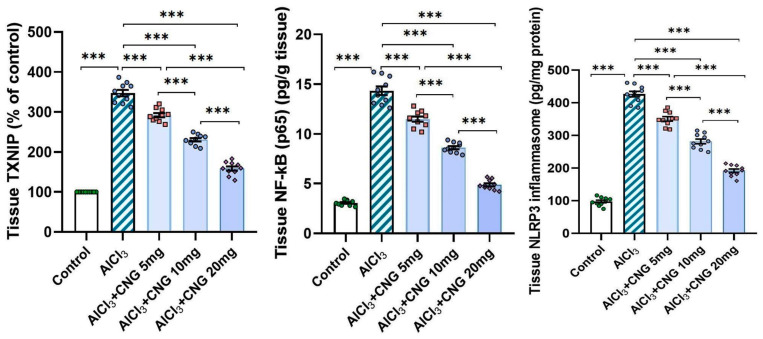
Effect of the different doses of canagliflozin on the hippocampal TXNIP/NF-κB p65/NLRP3 inflammasome signalling in rats injected with aluminium chloride (mean ± SD); *** = *p* < 0.001. AlCl_3_, aluminium chloride; CNG, canagliflozin.

**Figure 6:**
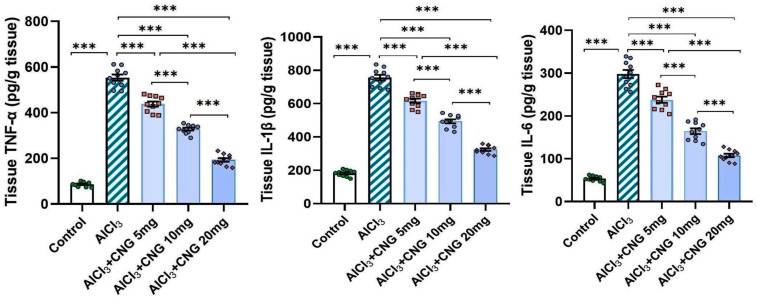
Effect of the different doses of canagliflozin on the hippocampal tissue levels of the pro-inflammatory cytokines in rats injected with aluminium chloride (mean ± SD); *** = *p* < 0.001. AlCl_3_, aluminium chloride; CNG, canagliflozin.

In the current study, aluminium chloride administration led to a significant elevation in the hippocampal tissue levels of TXNIP, NF-κB p65, and NLRP3 inflammasome together with induction of the inflammatory cascade manifested by a significant elevation in the hippocampal tissue levels of TNF-α, IL-1β, and IL-6 when compared to the control animals. Amazingly, canagliflozin in the present study exhibited a dose-dependent ability to reverse these biochemical alterations when compared to aluminium chloride-treated animals (Figure 5 and Figure 6).

### 3.4. Canagliflozin Dose-Dependently Mitigated the Effect of Aluminium Chloride Administration on SIRT1/HMGB1 Signalling in the Hippocampal Tissue Specimens

**Figure 7:**
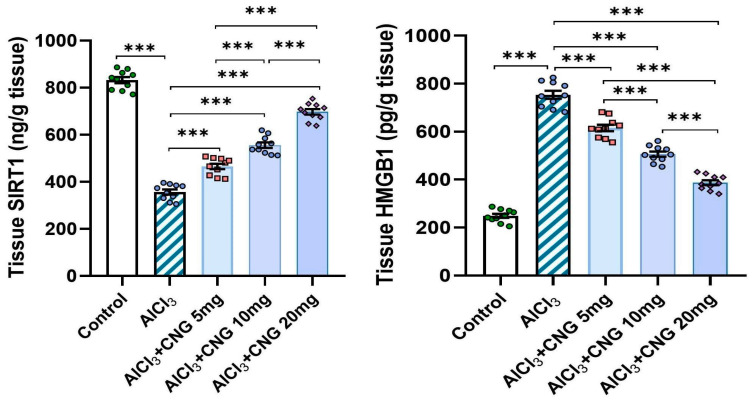
Effect of the different doses of canagliflozin on SIRT1/HMGB1 signalling in the hippocampal tissue specimens of rats injected with aluminium chloride (mean ± SD); *** = *p* < 0.001. AlCl_3_, aluminium chloride; CNG, canagliflozin.

As demonstrated in Figure 7, aluminium chloride was able to significantly decrease the hippocampal tissue levels of SIRT1 with concomitant elevation of the hippocampal tissue HMGB1 content relative to the control rats. These changes were significantly attenuated in the groups treated with canagliflozin, which showed a dose-dependent effect on these biochemical parameters.

### 3.5. Canagliflozin Dose-Dependently Augmented Autophagy in the Hippocampal Tissue Specimens of Animals Treated with Aluminium Chloride

**Figure 8:**
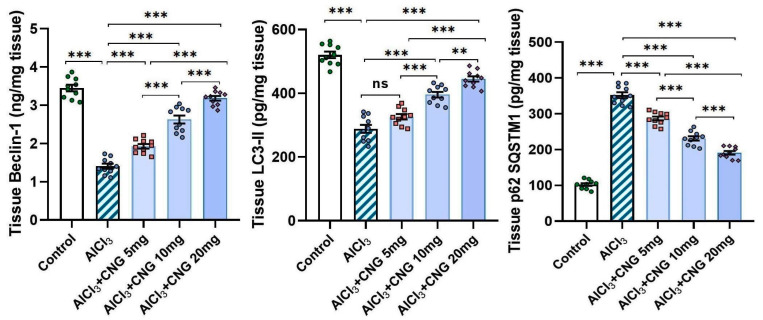
Effect of the different doses of canagliflozin on the autophagy markers in the hippocampal tissue specimens of rats injected with aluminium chloride (mean ± SD); ns = non-significant, ** = *p* < 0.01, *** = *p* < 0.001. AlCl_3_, aluminium chloride; CNG, canagliflozin.

As depicted in Figure 8, aluminium chloride had the ability to suppress autophagy, as indicated by a significant decline in beclin-1 and LC3-II levels associated with a significant elevation of p62 SQSTM1 levels in the hippocampal tissues when compared to the control animals. These changes were mitigated with the administration of canagliflozin in a dose-dependent manner, as evidenced by a significant rise in beclin-1 and LC3-II levels and a significant decline in p62 SQSTM1 levels in the hippocampal tissues when compared to the animals treated with aluminium chloride alone.

### 3.6. Canagliflozin Produced a Dose-Dependent Decline in the Hippocampal Tissue Levels of Caspase 3 and Bax in Aluminium Chloride-Treated Animals

**Figure 9:**
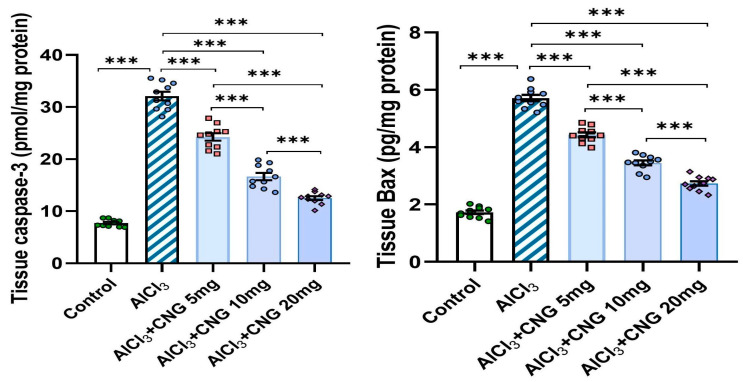
Effect of the different doses of canagliflozin on caspase 3 and Bax levels in the hippocampal tissue specimens of rats injected with aluminium chloride (mean ± SD); *** = *p* < 0.001. AlCl_3_, aluminium chloride; CNG, canagliflozin.

As demonstrated in Figure 9, aluminium chloride administration initiated apoptotic events, as indicated by a significant rise in the hippocampal tissue caspase 3 and Bax levels when compared to the findings of the control group. Interestingly, canagliflozin dose-dependently induced a significant decrement in the hippocampal tissue levels of caspase 3 and Bax when compared to the findings encountered in the animals treated with aluminium chloride alone.

### 3.7. Canagliflozin Dose-Dependently Ameliorated the Perturbations of the Mitochondrial Functions Induced by Aluminium Chloride in the Hippocampal Tissues

**Figure 10:**
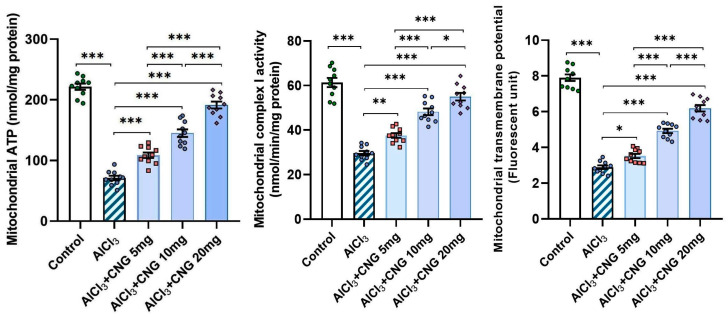
Effect of the different doses of canagliflozin on the mitochondrial ATP levels, mitochondrial complex I activity, and mitochondrial transmembrane potential in the hippocampal tissue specimens of rats injected with aluminium chloride (mean ± SD); * = *p* < 0.05, ** = *p* < 0.01, *** = *p* < 0.001. AlCl_3_, aluminium chloride; CNG, canagliflozin.

As illustrated in Figure 10, aluminium chloride administration elicited a state of mitochondrial dysfunction, as evidenced by a significant decrease in the mitochondrial ATP levels, mitochondrial complex I activity, and mitochondrial transmembrane potential relative to the control group. Meanwhile, treatment with canagliflozin led to a dose-dependent improvement in mitochondrial functions, as proven by the restoration of the mitochondrial ATP levels, mitochondrial complex I activity, and mitochondrial transmembrane potential to approximate the control values.

### 3.8. Canagliflozin Dose-Dependently Mitigated the Histopathological Changes in the Hippocampal Tissues Created as a Result of Aluminium Chloride Administration

**Figure 11:**
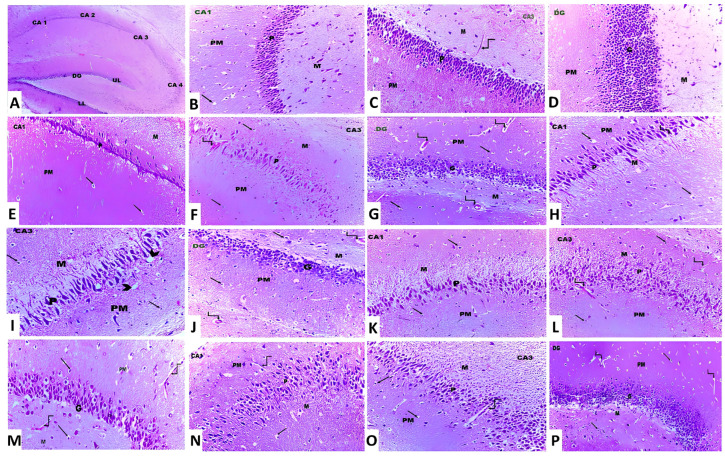
Photomicrographs of H&E-stained sections in the hippocampus from (**A**) the control group showing cornu ammonis, which is formed of CA1, CA2, CA3 and CA4. The dentate gyrus (DG) has an upper limb (UL) and a lower limb (LL) (×10). (**B**,**C**) The control group showing cornu ammonis with its three layers, namely polymorphic (PM), pyramidal (P) and molecular layer (M). The neuroglial cells are present in PM and M layers (arrow). Also, blood capillaries are seen within the molecular layer (curved arrow) (×20). (**D**) The control group showing the dentate gyrus, which is formed of molecular (M), granular (G) and polymorphic (PM) layers (×20). (**E**–**G**) Aluminium chloride-injected group, showing decreased thickness of the pyramidal (P) layer in CA1 and CA3 and the granular layer (G) in DG compared to the control group. Dilated and congested blood vessels (curved arrows) are also observed (×40). (**H**–**J**) Aluminium chloride group treated with canagliflozin, 5 mg/kg, showing a slight increase in the thickness of the pyramidal layer (P) in CA1 and CA3 and the granular layer (G) in DG compared to the group treated with aluminium chloride alone. The neuroglial cells (arrows) show perinuclear vacuolations and perinuclear halos (arrowhead). Dilated and congested blood vessels (curved arrows) are also observed (×40). (**K**–**M**) Aluminium chloride group treated with canagliflozin, 10 mg/kg, showing an obvious increase in the thickness of the pyramidal (P) layer in CA1 and CA3 and the granular layer (G) in DG compared to groups II and III. Dilated and congested blood vessels (curved arrows) are also observed (×40). (**N**–**P**) Aluminium chloride group treated with canagliflozin, 20 mg/kg, showing nearly complete restoration of the thickness of the pyramidal (P) layer in CA1 and CA3 and the granular layer (G) in DG as in the control group. Still dilated blood vessels (curved arrows) are also observed (×40).

In the present study, treatment of animals with aluminium chloride elicited a state of massive neurodegeneration associated with microglial activation and a significant decrement in the viable neurons when compared to the histopathological picture of the control animals. In contrast, treatment of aluminium chloride-injected rats with canagliflozin led to a dose-dependent significant mitigation of the histopathological changes with a significant rise in the number of normal polygonal neurons and a significant decrement in the number of dystrophic neuronal cells relative to the animals that received aluminium chloride alone (Figure 11).

### 3.9. Canagliflozin Dose-Dependently Augmented BCL-2 Immuno-Expression in the Hippocampal Tissues Induced by Aluminium Chloride

**Figure 12:**
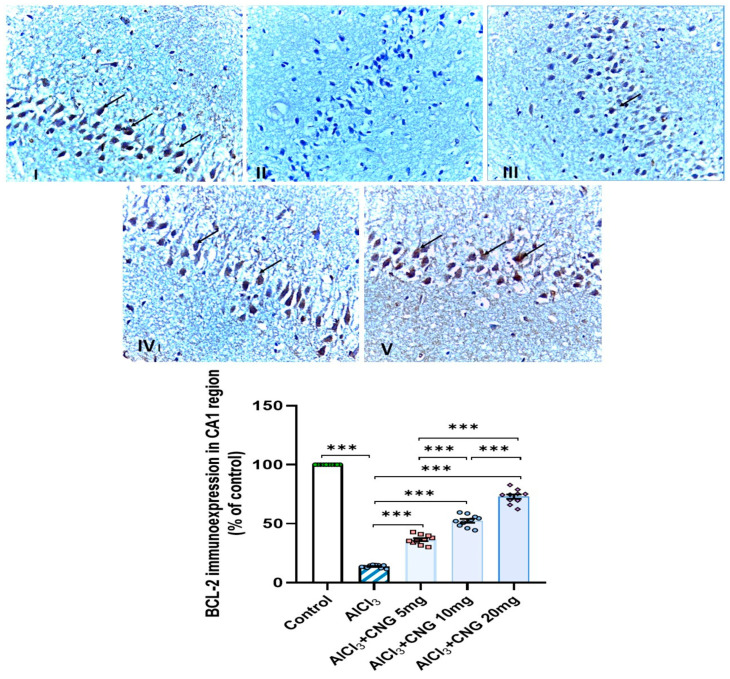
Photomicrographs representing the effect of the different treatments on the immune expression of BCL-2 in the hippocampal tissue specimens from the CA1 region of the studied groups. There is a significant decrease in the cytoplasmic immuno-expression of BCL-2 in the pyramidal cells in group **II** (aluminium chloride group). Groups **III** and **IV** treated with 5 mg/kg/day and 10 mg/kg/day canagliflozin, respectively, show moderate immuno-expression of BCL-2. In both groups **I** (control) and **V** (treated with 20 mg/kg/day canagliflozin), there is strong immunoreaction (BCL-2 immunostaining, ×40). *** = *p* < 0.001. AlCl_3_, aluminium chloride; CNG, canagliflozin.

**Figure 13:**
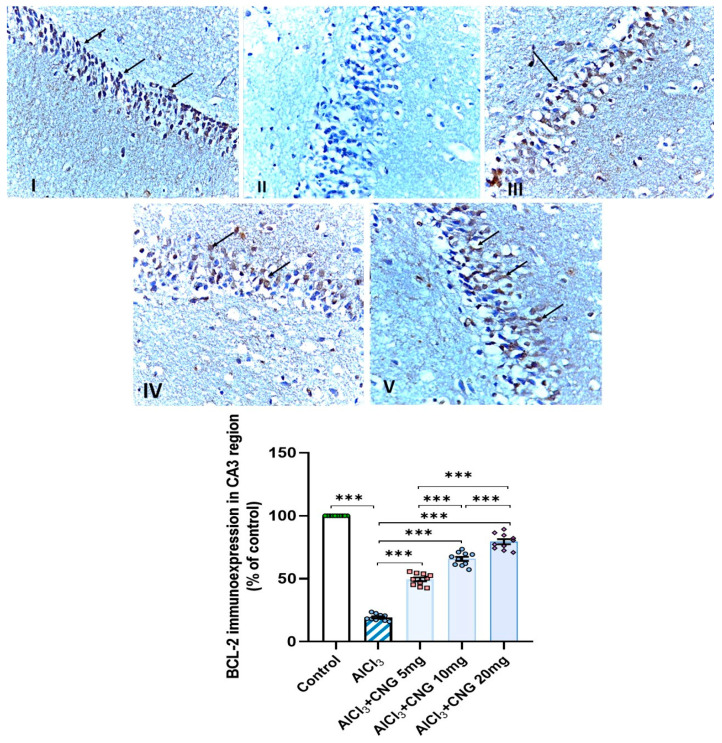
Photomicrographs representing the effect of the different treatments on the immuno-expression of BCL-2 in the hippocampal tissue specimens from the CA3 region of the studied groups. There is a significant decrease in the cytoplasmic immuno-expression of BCL-2 in the pyramidal cells in group **II** (aluminium chloride group). Groups **III** and **IV** treated with 5 mg/kg/day and 10 mg/kg/day canagliflozin, respectively, show moderate immuno-expression of BCL-2. In both groups **I** (control) and **V** (treated with 20 mg/kg/day canagliflozin), there is strong immunoreaction (BCL-2 immunostaining, ×40). *** = *p* < 0.001. AlCl_3_, aluminium chloride; CNG, canagliflozin.

**Figure 14:**
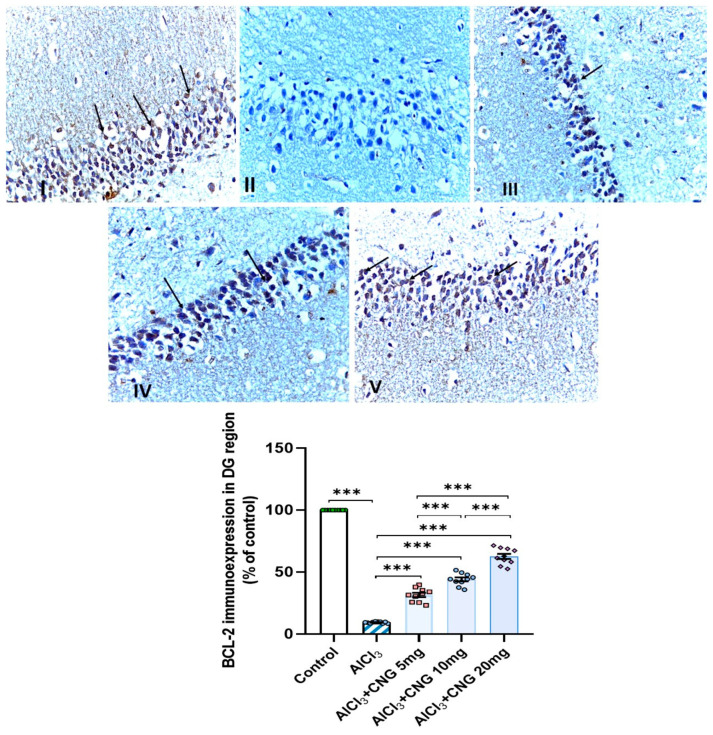
Photomicrographs representing the effect of the different treatments on the immuno-expression of BCL-2 in the hippocampal tissue specimens from the DG region of the studied groups. There is a significant decrease in the cytoplasmic immuno-expression of BCL-2 in the granular cells in group **II** (aluminium chloride group). Groups **III** and **IV** treated with 5 mg/kg/day and 10 mg/kg/day canagliflozin, respectively, show moderate immuno-expression of BCL-2. In both groups **I** (control) and **V** (treated with 20 mg/kg/day canagliflozin), there is strong immunoreaction (BCL-2 immunostaining, ×40). *** = *p* < 0.001. AlCl_3_, aluminium chloride; CNG, canagliflozin.

As demonstrated in Figure 12, Figure 13 and Figure 14, aluminium chloride injection was associated with a significant decline in BCL-2 immunostaining of the hippocampal tissue specimens when compared to the control group. These changes were significantly attenuated with administration of the different doses of canagliflozin to aluminium chloride-treated animals, with the most favourable responses being recorded in the group that received 20 mg/kg/day of canagliflozin.

## 4. Discussion

Alzheimer’s disease (AD) represents a chronic disabling neurological disorder that results from neurodegeneration that may eventually lead to significant effects on the memory and cognition of patients, with a negative impact on their quality of life [25]. Several research projects that were conducted in recent decades concluded that no single mechanism can be considered to be responsible for the pathogenesis of this disorder [26]. Perturbations of the antioxidant defences and augmentation of the inflammatory cascade in the central nervous system with subsequent effects on the balance between autophagy and the apoptotic signals were postulated as possible contributing factors [27]. Aluminium chloride is often used in experimental research to induce AD in animals [28]. The importance of this model is attributed to the findings that it induces several key features that resemble AD in humans including cognitive impairment, oxidative tissue damage, neuroinflammation, and formation of amyloid beta plaques [29]. In addition, exposure to aluminium chloride leads to hyperphosphorylation of tau proteins, leading to the formation of neurofibrillary tangles which interfere with the normal functions of the neurons, leading to neuronal cell death, which is the hallmark of AD [30]. Interestingly, ROS produced in excess as a result of aluminium chloride exposure were reported to enhance misfolding and fragmentation of tau proteins, with the net result of exacerbation of the formation of the neurofibrillary tangles [31]. These mechanisms were revealed in the current work, where injection of aluminium chloride induced serious perturbation of memory functions manifested as a statistically significant decrease in the discrimination index and the sniffing time relative to the control group. Additionally, the time taken to reach a hidden platform was significantly prolonged and the time and the distance spent in the target quadrant were significantly shortened in aluminium chloride-treated rats when compared to those taken by the control group.

Nrf2 signalling and its downstream products have been proven to play an outstanding role in the pathogenesis of neurodegenerative disorders, including AD [32]. In comparison to normal subjects, patients with neurodegenerative disorders recorded significantly lower levels of Nrf2 in their brain tissues, which were accompanied by a significant deterioration in the tissue antioxidant defence mechanisms and were directly proportional to the extent of neurodegeneration [33]. This may be due to the inhibitory effects of ROS on Nrf2 production with subsequent effects on both the genetic expression and the activity of the antioxidant enzymes, with the end result of significant deterioration in the histomorphic features of the brain tissues [34]. This confirmed the findings of the current study, where rats injected with aluminium chloride exhibited a statistically significant decline in Nrf2 content, antioxidant enzymes, and TAC of the hippocampal tissues associated with a significant rise in the tissue levels of MDA relative to the control rats which did not receive aluminium chloride injection. Interestingly, treatment with canagliflozin in the present study showed an outstanding ability to ameliorate these changes, which may be attributed to enhancement of Nrf2-dependent signalling pathways with subsequent potentiation of the antioxidant defence mechanisms and reinforcement of the free radical scavenging activities in the brain tissues [6].

TXNIP is a glucocorticoid-regulated primary response gene that is thought to mediate a number of signalling pathways that regulate the different cellular processes in the human body [35]. Recent reports have drawn attention towards the role of TXNIP in the pathogenesis of neurodegenerative disorders, including AD [36]. Pan et al. [37] stated that TXNIP represents a joining link between redox reactions and apoptosis. Increased production of ROS in AD was proven to increase the genetic expression of TXNIP with subsequent activation of the apoptotic pathways [38]. These reports confirmed the findings of the current work, where aluminium chloride injection was associated with a significant elevation of TXNIP levels in the hippocampal tissues together with significant interference with the antioxidant defence mechanisms and significantly elevated levels of the apoptotic mediators when compared to the results encountered with the control group. Moreover, TXNIP was proven to be a key mediator in the cellular inflammatory responses of the hippocampal tissues to aluminium chloride injection, as demonstrated in the present study [39]. This may be attributed to the findings that aluminium chloride activates the TXNIP/NLRP3 inflammasome axis, which in turn enhances the production of pro-inflammatory cytokines and induces massive inflammatory infiltration of the hippocampal tissues [40]. Additionally, TXNIP was reported to directly enhance NF-κB transcription, which is the cornerstone of the inflammatory events that are pathognomonic for neurodegenerative disorders, including AD [41].

In the present study, aluminium chloride-injected animals treated with different doses of canagliflozin had significantly lower levels of TXNIP, NLRP3 inflammasome, NF-κB, and pro-inflammatory cytokines relative to animals that received aluminium chloride alone. This may be due to the modulatory effects of SGLT2 inhibitors, including canagliflozin, on TXNIP/NLRP3 signalling with subsequent amelioration of neuroinflammation [42]. In addition, the modulatory effects of canagliflozin on toll-like receptor 4/NLRP3 inflammasome signalling might potentiate its anti-inflammatory effects in various neurological disorders [43]. Moreover, the dose-dependent inhibitory effect of canagliflozin on the apoptotic pathways that was detected in the present study may originate from the ability of SGLT2 inhibitors to repress TXNIP production, which deprives the apoptotic cascade of one of the most important apoptosis enhancers [44]. Additionally, canagliflozin-mediated inhibition of the mammalian target of rapamycin (mTOR) and NF-κB signalling might add value to its antiapoptotic effects, regardless of its hypoglycaemic influences [45].

SIRT1 is a nicotinamide adenine dinucleotide (NAD)-dependent deacetylase that mediates mammalian metabolic responses in the different tissues of the body [46]. A strong relationship was found between the hippocampal SIRT1 levels and the pathogenic events that are characteristic of AD [47]. It was reported that SIRT1 has an outstanding ability to abrogate oxidative stress in the hippocampal tissues through augmentation of the expression of peroxisome proliferator-activated receptor-γ co-activator 1α (PGC-1α) with significant suppression of ROS production [48]. Moreover, SIRT1 modulates the signalling pathways in which FoxO1 plays a crucial role, thereby increasing the production and activity of the antioxidant enzymes [49]. Additionally, SIRT1 was proven to suppress the activity of a number of nuclear proteins that are responsible for the regulation of the inflammatory responses in the hippocampal tissues [50]. Among these proteins, HMGB1 emerges as a ubiquitous nuclear protein that interacts with various target cell receptors, including the receptors for advanced glycation end-products (RAGEs) and toll-like receptor (TLR) 4, and increases the expression and release of pro-inflammatory cytokines, an action that is largely mediated via NLRP3 inflammasome/NF-κB signalling [51]. These events were clearly obvious in the present work, where aluminium chloride-mediated inhibition of SIRT1 signalling with subsequent increases in HMGB1 expression and augmentation of the inflammatory process and oxidative stress in the hippocampal tissues were significantly mitigated with the administration of different doses of canagliflozin. This may be attributed to the ability of SGLT2 inhibitors, including canagliflozin, to directly activate the expression of SIRT1 and repress nucleoplasmic translocation and the extracellular release of HMGB1 in the brain tissues [6,52].

Ongoing research efforts have established a causal relationship between mitochondrial dysfunction and the pathogenic features of aluminium chloride-induced AD [14]. In agreement with the findings of the present study, Chen et al. [53] reported that aluminium chloride-induced cognitive impairment and oxidative stress may involve impairment of the mitochondrial functions in the brain with significant decreases in mitochondrial ATP levels. Memudu and Adanike [54] indicated that the mitochondria of the neuronal cells and the astrocytes act in harmony to maintain neuronal homeostasis. Interference with the mitochondrial functions as a result of aluminium chloride administration will be reflected by increased matrix production by astrocytes as a consequence of ROS release [49]. This in turn initiates cellular events characterised by activation of caspases, leading to progressive neuronal cell death [55]. In addition, aluminium chloride-induced mitochondrial dysfunction may facilitate gliosis as a response to profound neuroinflammation, leading to significant cognitive impairment [54]. An interesting finding in the current work was the significant improvement of the mitochondrial functions in aluminium chloride-injected groups treated with canagliflozin, manifested by a significant increase in the mitochondrial ATP levels, complex I activity, and the mitochondrial transmembrane potential in comparison to the same parameters in the group that received aluminium chloride alone. These findings were in accordance with Wang et al. [56] who reported that SGLT2 inhibitors, including canagliflozin, preserve the mitochondrial functions via different mechanisms, including enhancement of the mitochondrial calcium buffer capacity, prevention of mitochondrial swelling, and improvement of mitochondrial fission. Additionally, Dabravolski et al. [57] stated that STGL2 inhibitors maintain the structural integrity of the mitochondrial membranes, stimulate mitochondrial biogenesis, enhance mitochondrial ATP production, repress mitochondrial ROS generation, and decrease the levels of mitochondrial lipid peroxidation products. Moreover, the ability of SGLT2 inhibitors to enhance the activities of the mitochondrial complex I and complex III activities and reduce mitochondrial DNA damage may add value to the protective effect of canagliflozin against mitochondrial dysfunction [58].

Ongoing research has been directed towards exploration of the possible linkage between perturbations of autophagy and the characteristic neurodegenerative features of aluminium chloride-induced AD [59]. Raj et al. [60] reported that administration of aluminium chloride significantly interferes with the autophagic capacity of the brain tissues. Coinciding with the results encountered in the present work, Weng et al. [61] stated that aluminium chloride injection was associated with significantly lower levels of beclin-1 and LC3-II in the brain tissues relative to the control findings. Awad et al. [62] attributed aluminium chloride-mediated suppression of the autophagic flux to its ability to affect the PI3K/Akt/mTOR pathway with subsequent attenuation of the genetic expression of autophagy mediators. In addition, aluminium chloride-induced augmentation of TXNIP/NLRP3 inflammasome signalling was reported to combat autophagy responses, an action that is largely mediated through inhibition of the phagocytic functions of the microglial cells [40,63]. Amazingly, canagliflozin in the current study exhibited a dose-dependent outstanding ability to improve the autophagic signals together with significant decrements in p62 SQSTM1 levels, which is a potent autophagy suppressor protein, in the hippocampal tissues when compared to the group treated with aluminium chloride alone. These autophagy-promoting effects of canagliflozin may be attributed to its ability to suppress mTOR/NF-κB signalling and its downstream products, thus freeing the autophagy mechanisms from their suppressive effects [64]. Moreover, canagliflozin was proven to enhance AMPK/SIRT1 signalling with subsequent restoration of the autophagy markers and inhibition of the apoptotic pathways [65].

The major limitation of the present study is that it did not assess the potential safety of canagliflozin for cases of AD. This limitation originates from the chronic nature of AD, which necessitates the use of drugs for a long period of time, which increases the risk of adverse effects. Long-term use of canagliflozin was reported to be associated with significant decreases in bone mineral density and bone strength, which may interfere with the mechanical functions of bones [66]. In addition, it may increase the incidence of genital mycotic and urinary tract infections and their serious complications [15]. Moreover, the incidence of ketoacidosis was reported to increase with long-term use of canagliflozin, which may even endanger life [67]. These effects must be properly evaluated before the introduction of canagliflozin as a promising agent for the management of AD. Another limitation of the current study was the relatively small number of rats used and the relatively short duration of the study. Our future research plans will be designed to involve larger numbers of animals for longer periods of time to assess the long-term adverse effects of canagliflozin on various tissues of the body.

The application of the findings of the present study in the clinical management of AD in humans will be faced with a number of challenges. These include the biological variations between humans and rats, which may significantly affect the pharmacokinetics of canagliflozin. In addition, humans possess a high degree of genetic variability compared to rats. This variability may significantly affect the individual responses of patients to canagliflozin. Moreover, the controlled environmental circumstances in which the laboratory rats are kept do not accurately reflect the complex and highly variable nature of the environments surrounding humans which may significantly affect their individual responses to canagliflozin. Also, issues related to patients’ compliance to treatment with canagliflozin may represent additional challenges to the clinical application of the results of the present study.

## 5. Conclusions

**Figure 15:**
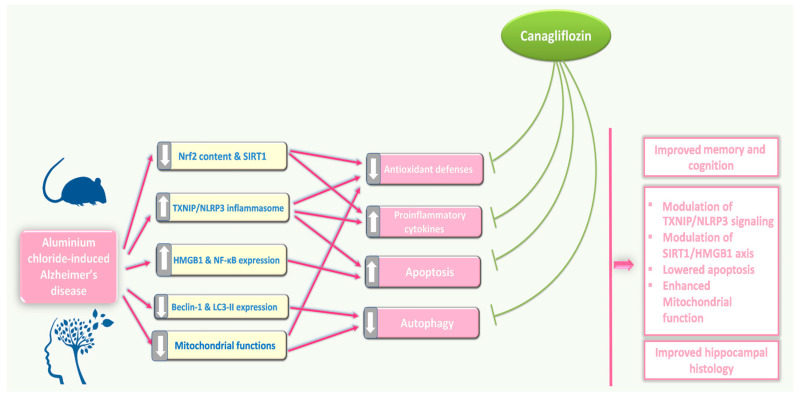
An illustrative diagram of the mechanisms by which the different doses of canagliflozin may mitigate the pathologic changes in the hippocampal tissues created by aluminium chloride injection.

Inhibition of TXNIP/NLRP3 inflammasome signalling, modulation of SIRT1/HMGB1 axis, interference with pro-inflammatory and pro-apoptotic mechanisms, and restoration of mitochondrial functions and autophagy may represent possible effects by which canagliflozin may emerge as a promising agent for management of the features of AD (Figure 15). Future research efforts should be directed towards a more in-depth investigation of the effects of different doses of canagliflozin on these mechanisms and to provide a reliable evaluation of the benefits of its clinical application.

## Data Availability

The data are available from the corresponding author upon reasonable request.
